# Viburnum, or Black Haw

**Published:** 1875-12

**Authors:** John J. Addy

**Affiliations:** Meriwether County, Ga.


### VIBURNUM, OR BLACK HAW.

By JOHN J. ADDY, M.D., of Meriwether County, Ga.

The habitat of the viburnum is variable in different localities. Whilst for the most part it is to be found upon the banks of streams and in the low lands, in certain regions it grows alone upon the highest elevations.

In the year 1861 or '62, my attention was first called to this remedy by one outside the profession, who spoke highly of its powers as a uterine sedative. From that time to this I have used it very uniformly in my practice, and with most satisfactory results. I have used the viburnum alone, and at times in conjunction with opium. In some instances I have known it to succeed after opium had failed to arrest the uterine action. In one case I began the use of opium at 11 A.M., and by 3 P.M. had given seven grains of opium without controlling the uterine pains. I now had the black haw procured and made into strong decoction, of which a teacupful was administered. In one hour the pains ceased, and did not return. In a second case, the pains had been going on for twenty-four hours; the strong decoction was given in dose of two and a half ounces, and the same repeated in thirty minutes. In three hours' time the pains ceased, and the patient went to full term. It has been common for me to recommend my patients thus threatened to keep the viburnum by them and use the decoction as needed.

Viburnum seems to exercise a specific action upon the muscular stucture of the uterus through the medium of the excito-motor system of nerves, just as ergot is supposed to do, but in the opposite direction. I esteem it to be a direct uterine sedative.

Aside from its use as an anti-abortifacient, I have employed it also in the treatment of hysteria in many cases, and with favorable results. Its efficacy in this malady is likely due to its specific action upon the uterus. I use it always in strong decoction or infusion of the root, and in doses varying from two to six ounces. The dose is repeated every hour or two until the symptoms are relieved. The remedy is innocent of any unpleasant effects upon the system, and may be given *ad libitum.*

